# Supplementary material for: Extremely low-frequency pulses of faint magnetic field induce mitophagy to rejuvenate mitochondria
Source: Commun Biol. 2022 May 12;5:453. doi: 10.1038/s42003-022-03389-7 (PMC9098439; doi:10.1038/s42003-022-03389-7)

Supplementary information for

Extremely low-frequency pulses of faint magnetic field induce mitophagy to rejuvenate mitochondria

Takuro Toda[1], Mikako Ito[1], Jun-ichi Takeda[1], Akio Masuda[1], Hiroyuki Mino[2], Nobutaka Hattori[3], Kaneo Mohri[4], Kinji Ohno[1*]

[1]Division of Neurogenetics, Center for Neurological Diseases and Cancer, Nagoya University Graduate School of Medicine, Nagoya, Japan
[2]Division of Material Science, Nagoya University Graduate School of Science, Nagoya, Japan
[3]Department of Neurology, Juntendo University, Tokyo, Japan
[4]Nagoya Industrial Science Research Institute, Nagoya, Japan

This PDF file includes:

Supplementary Table 1, 2

Supplementary Figure 1-6

Supplementary Table1. List of abbreviations

Abbreviation	Definition
WMF	weak magnetic fields
ELF	extremely low-frequency
ETC	electron transport chain
ROS	reactive oxygen species
PINK1	PTEN-induced kinase 1
TMRM	Tetramethylrhodamine, Methyl Ester, Perchlorate
OCR	oxygen consumption rate
DCPIP	2, 6-dichlorophenolindophenol
SDH	succinate dehydrogenase
SQR	succinate:quinone reductase
SCR	succinate cytochrome *c* reductase
SDHA	succinate dehydrogenase [ubiquinone] flavoprotein subunit
SDHB	succinate dehydrogenase [ubiquinone] iron-sulfur subunit
SDHC	succinate dehydrogenase cytochrome b560 subunit
SDHD	succinate dehydrogenase [ubiquinone] cytochrome b small subunit

Supplementary Table 2. The effects of Opti-ELF-WMF on mitochondrial mass and mitochondrial membrane potential in six cell lines

Cells	Mitochondrial mass (MitoTracker Green) at 3 h (%)	Mitochondrial membrane potential (TMRM) at 12 h (%)
AML12	$73.42 \pm 2.26^{**}$	$110.43 \pm 4.15^{*}$
Neuro2a	$75.68 \pm 3.11^{**}$	104.86 ± 4.31
C2C12	$67.96 \pm 1.31^{****}$	$106.95 \pm 1.79^{*}$
Human iPS	$84.65 \pm 2.76^{**}$	$116.66 \pm 1.33^{*}$
HEK	$82.55 \pm 3.27^{***}$	106.36 ± 6.35
HeLa	$84.06 \pm 5.15^{**}$	$102.85 \pm 0.39^{*}$

Mean and SD are indicated (n = 4 dishes each; $^{*}q < 0.05$, ** $q < 0.01$, *** $q < 0.001$, *** $q < 0.001$ by multiple Student's t-tests).

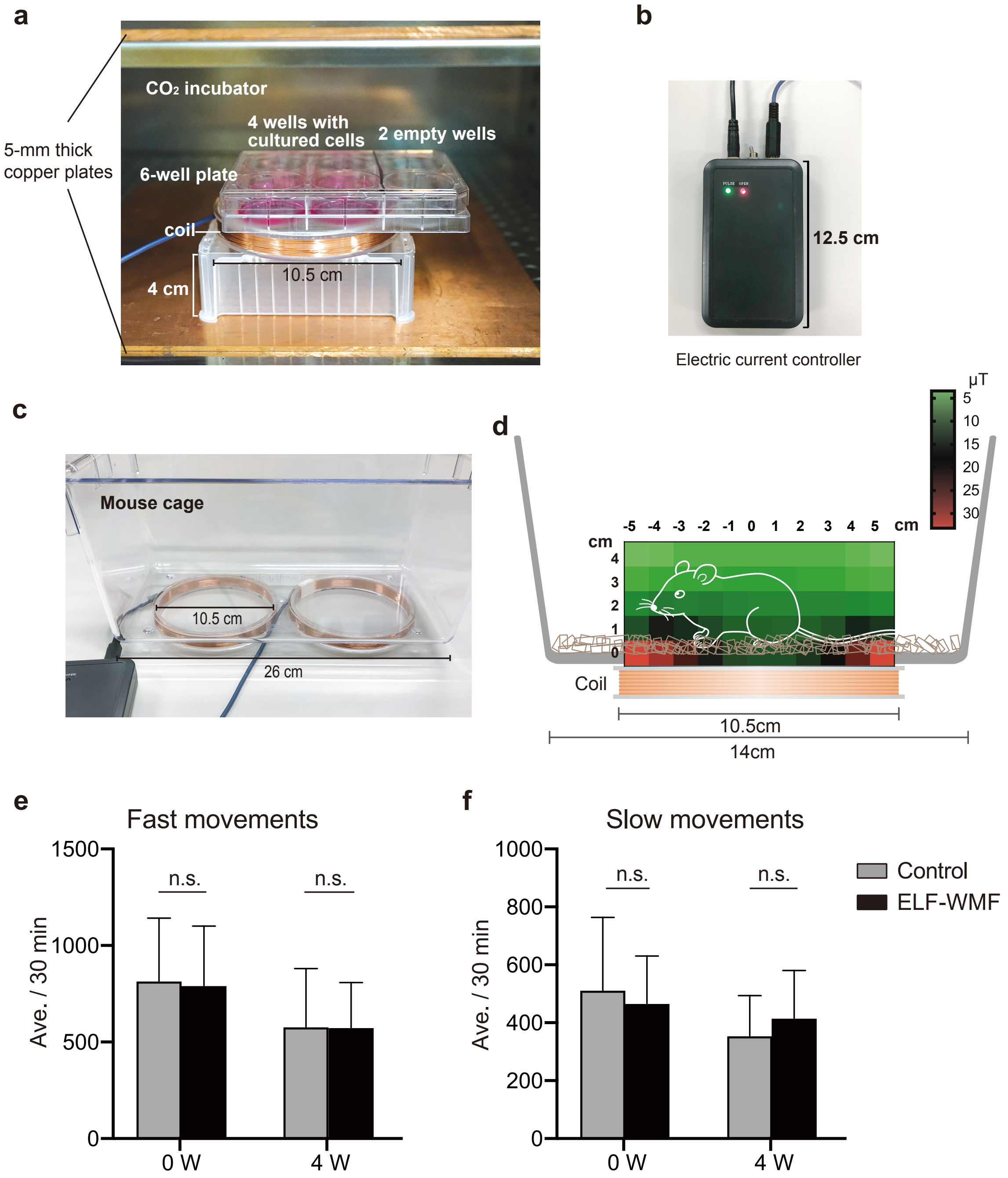

Supplementary Figure 1. The setup of the ELF-WMF device and lack of the effect of Opti-ELF-WMF for 4 weeks on the open-field locomotor activities in wild-type mice, related to Figure 1.

a ELF-WMF setup for cultured cell. Four of the six wells were used for cell culture. All cells were fit within a 10.5-cm coil. Cells were sandwiched by copper plates, and the coil was placed 4 cm above the bottom plate. Current was applied clockwise when viewed from the top. Cells were cultured ~1 cm above the top of the coil, and the magnetic field

intensities can be referred to the color coding in **d**. **b** Electric current controller for the ELF-WMF coil. **c** Two ELF-WMF coils were placed directly beneath a cage for mouse studies. Current was applied clockwise for both coils when viewed from the top. **d** Color coding of measured magnetic field intensities in a mouse cage above a coil. Exposure to Opti-ELF-WMF for 4 weeks had no effect on fast **(e)** and slow **(f)** movements of wild-type mice as determined by an open-field locomotor activity test (mean ± SD, $n = 14$ mice each; no statistical [n.s.] difference by Kruskal–Wallis test).

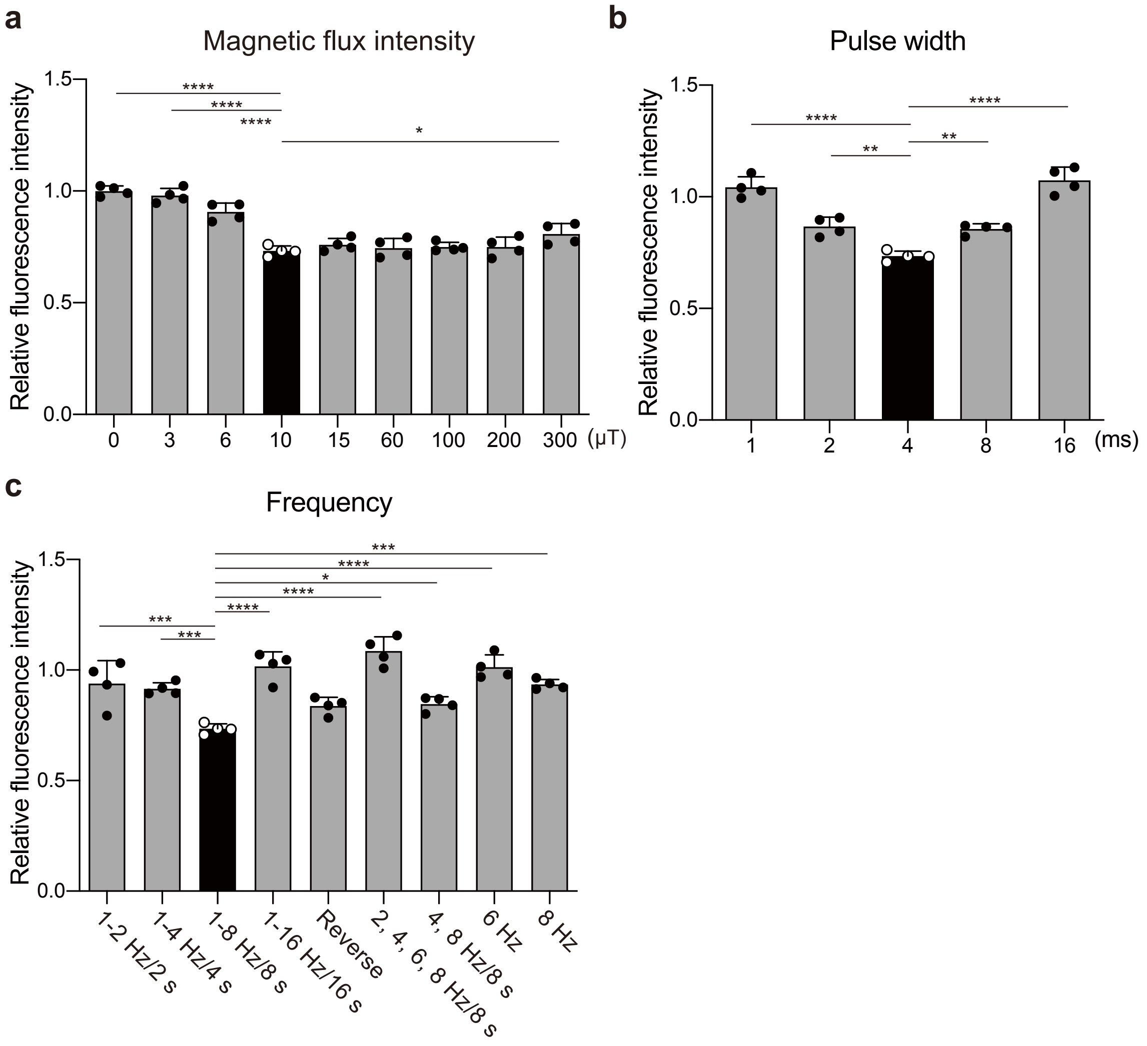

Supplementary Figure 2. Optimization of ELF-WMF.

Optimization of magnetic flux intensity **(a)**, pulse width **(b)**, and pulse frequency **(c)** of ELF-WMF to reduce the mitochondrial mass (MitoTracker Green) of AML12 cells in 3 h (mean ± SD, n = 4 culture dishes each; $^{*}p < 0.05$, $^{**}p < 0.01$, $^{***}p < 0.001$, and $^{****}p < 0.0001$ by one-way ANOVA followed by Dunnett's posthoc test between the black bar and the gray bars). Conditions of Opti-ELF-WMF are indicted by black bars.

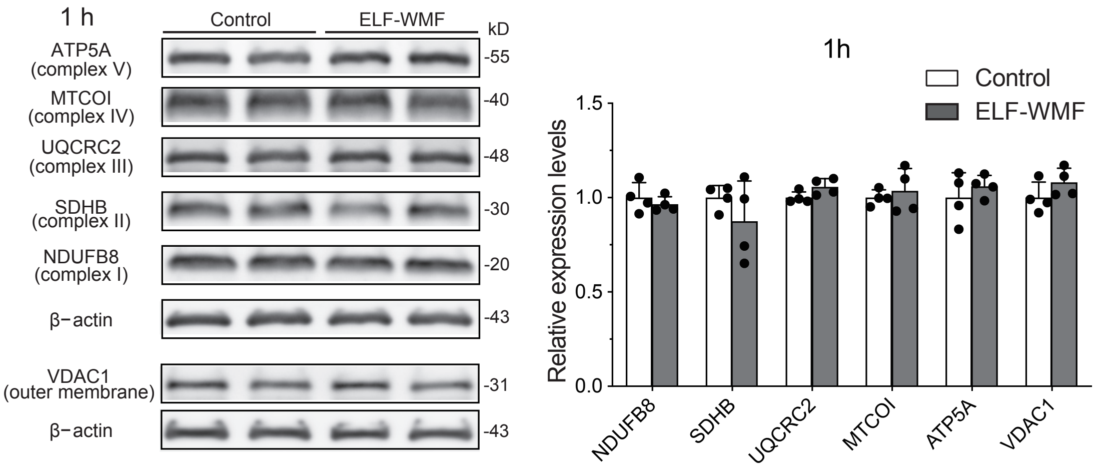

Supplementary Figure 3. Exposure to Opti-ELF-WMF for 1 h did not change the amounts of mitochondrial proteins in AML12 cells, related to Figure 3.

Western blot analysis of mitochondrial oxidative phosphorylation proteins and a mitochondrial outer membrane protein, VDAC1, in AML12 cells exposed to Opti-ELF-WMF for 1 h (mean ± SD, n = 4 culture dishes each; no statistical difference by multiple Student's t-tests).

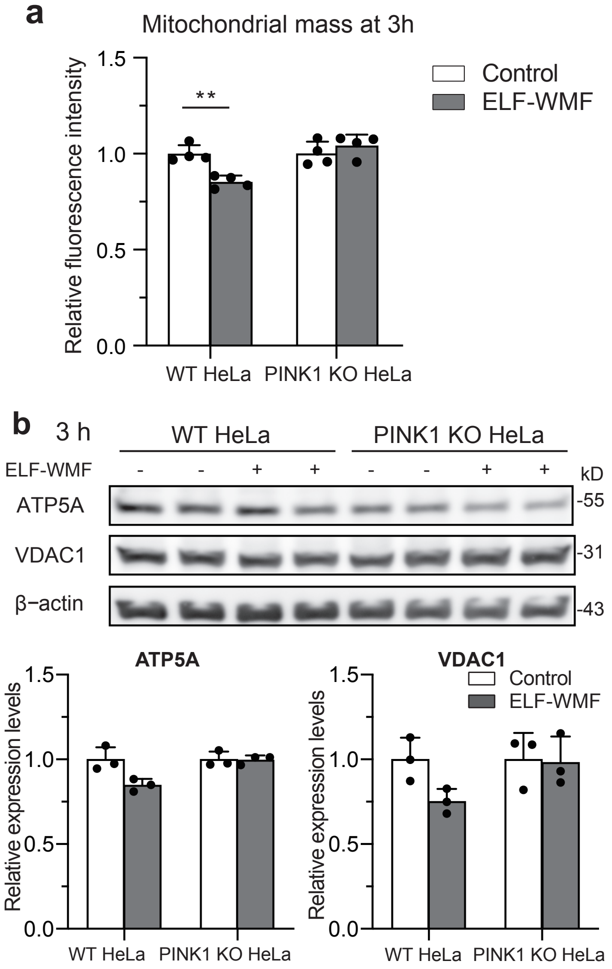

Supplementary Figure 4. Opti-ELF-WMF failed to induce mitophagy at 3 h in PINK1-kocked out (KO) HeLa cells, related to Figure 4.

a Mitochondrial mass (MitoTracker Green) of wild-type (WT) and PINK1 KO HeLa cells exposed to Opti-ELF-WMF for 3 h (mean ± SD, *n* = 4 dishes each; $^{**}q < 0.01$ by multiple Student's *t*-tests). **b** Representative duplicates of western blot analysis for ATP5A and VDAC1 in WT and PINK1 KO HeLa cells incubated under Opti-ELF-WMF for 3 h (mean ± SD, *n* = 4 dishes each; no statistical difference by multiple Student's *t*-tests).

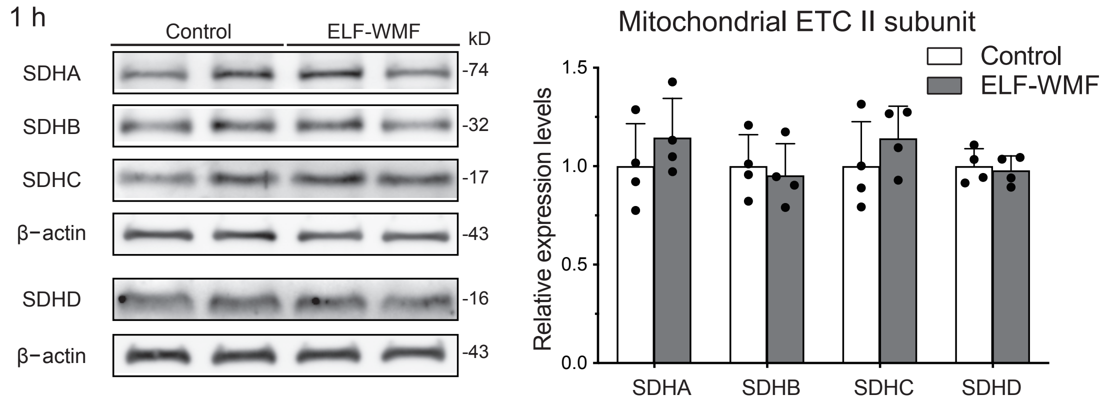

Supplementary Figure 5. Exposure to Opti-ELF-WMF for 1 h had no effects on the amount of mitochondrial ETC II subunits in AML12 cells, related to Figure 6. Representative duplicates of western blot analysis for mitochondrial ETC II subunits of AML12 cells exposed to Opti-ELF-WMF for 1 h (mean ± SD, *n* = 4 dishes each; no statistical difference was observed by multiple Student's *t*-tests).

Supplementary Figure 6. Original blot images

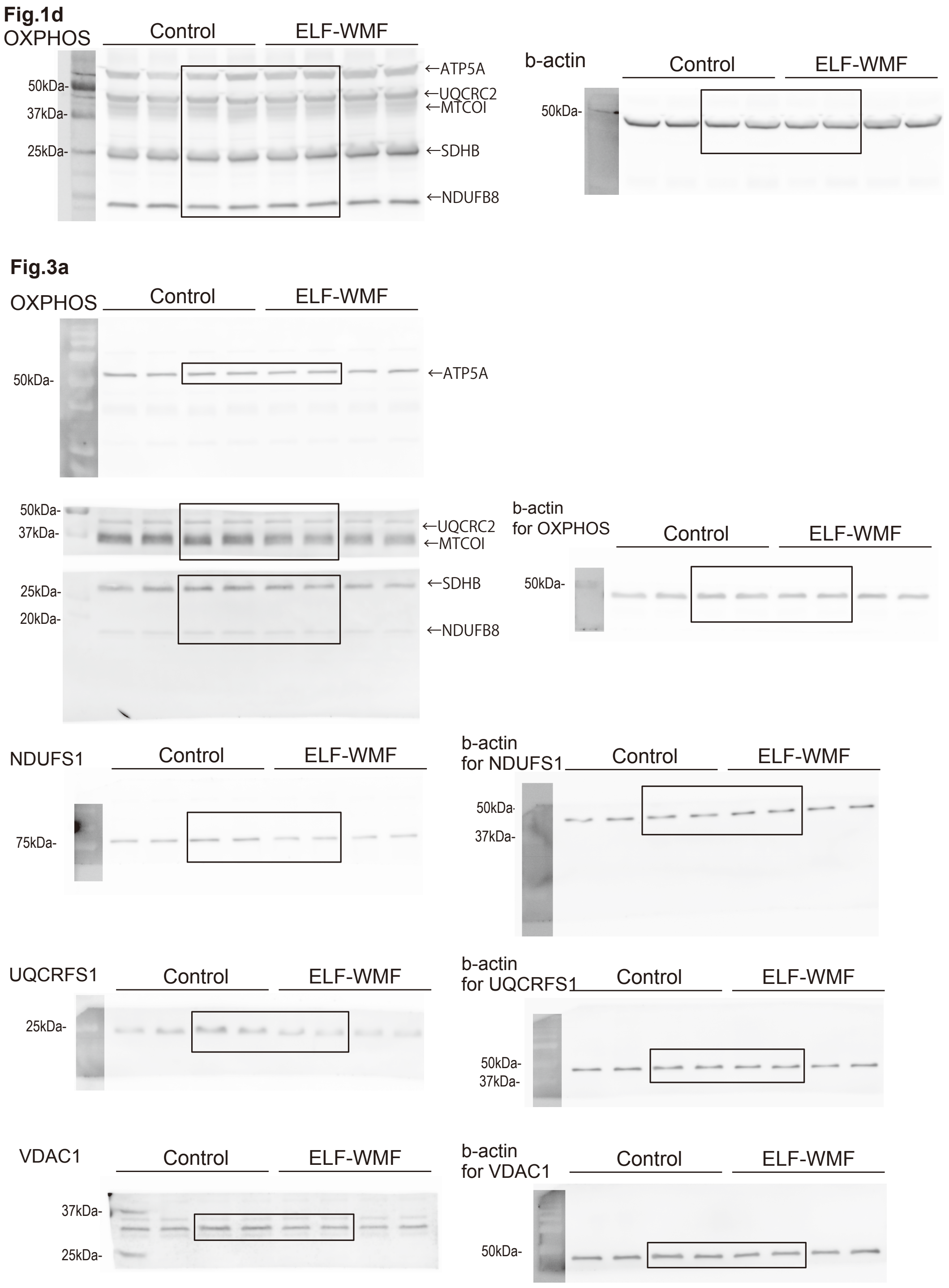

Fig.3b

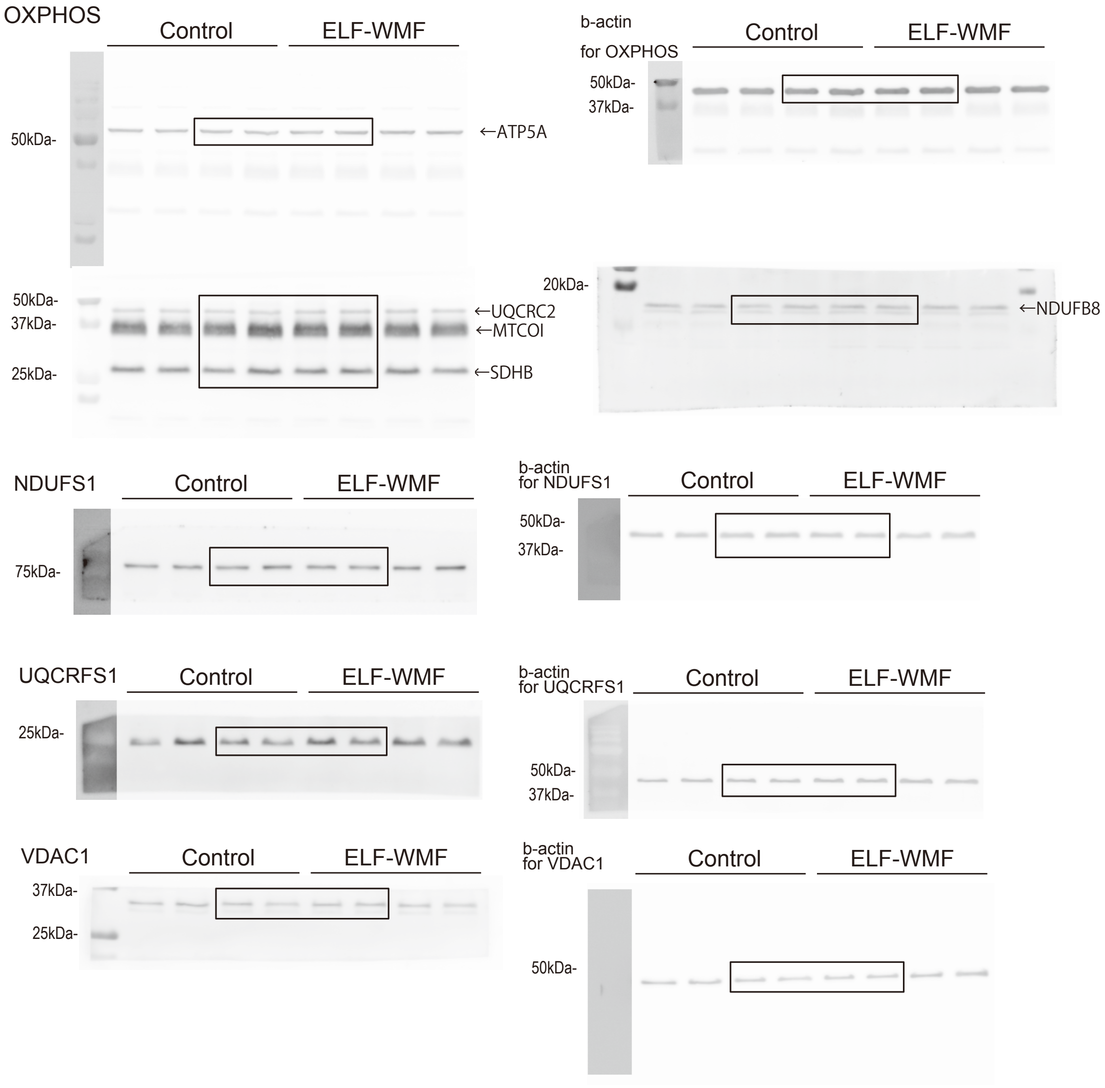

OXPHOS
Control
ELF-WMF
50kDa-
←ATP5A
50kDa-
37kDa-
25kDa-
←UQCRC2
←MTCOI
←SDHB
b-actin
for OXPHOS
Control
ELF-WMF
50kDa-
37kDa-
20kDa-
←NDUFB8
NDUFS1
Control
ELF-WMF
75kDa-
b-actin
for NDUFS1
Control
ELF-WMF
50kDa-
37kDa-
UQCRFS1
Control
ELF-WMF
25kDa-
b-actin
for UQCRFS1
Control
ELF-WMF
50kDa-
37kDa-
VDAC1
Control
ELF-WMF
37kDa-
25kDa-
b-actin
for VDAC1
Control
ELF-WMF
50kDa-

Fig.4a

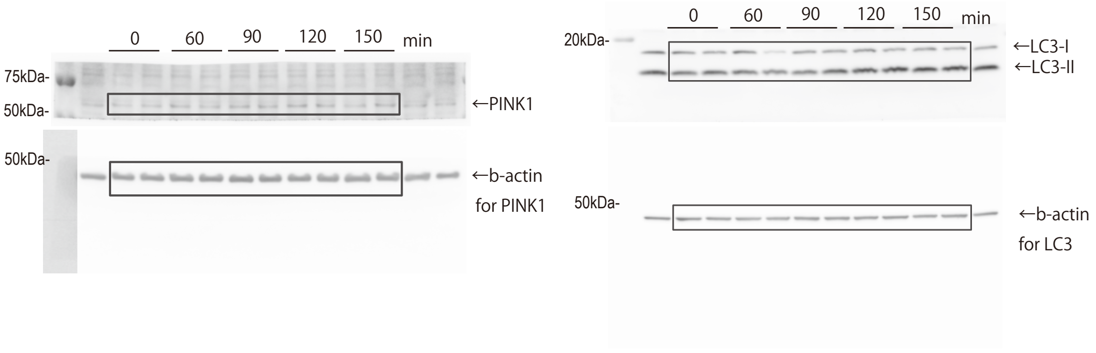

0
60
90
120
150
min
75kDa-
50kDa-
←PINK1
50kDa-
←b-actin
for PINK1
20kDa-
←LC3-I
←LC3-II
50kDa-
←b-actin
for LC3

Fig.4b Whole

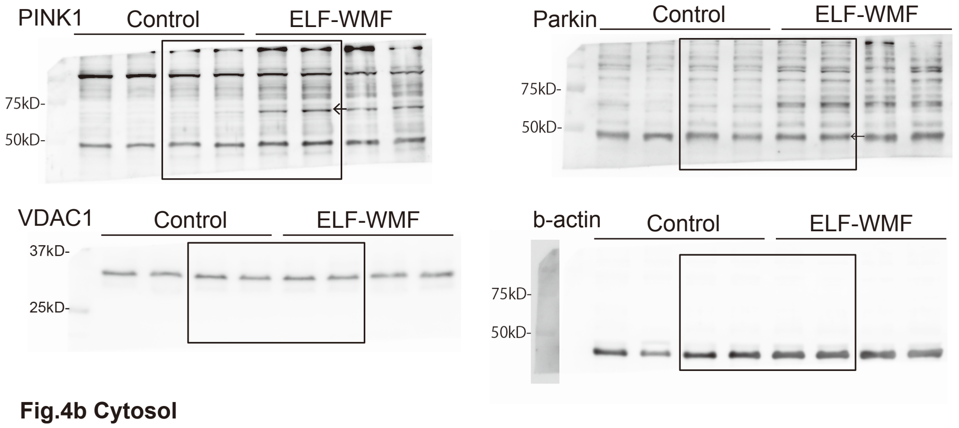

Fig.4b Cytosol

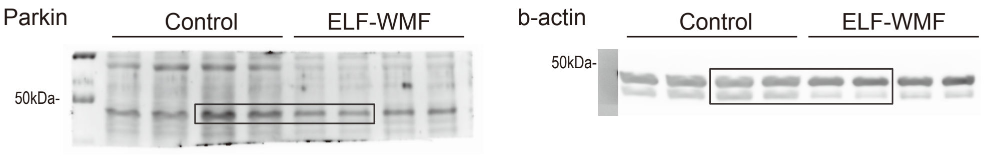

Fig.4b Mitochondria

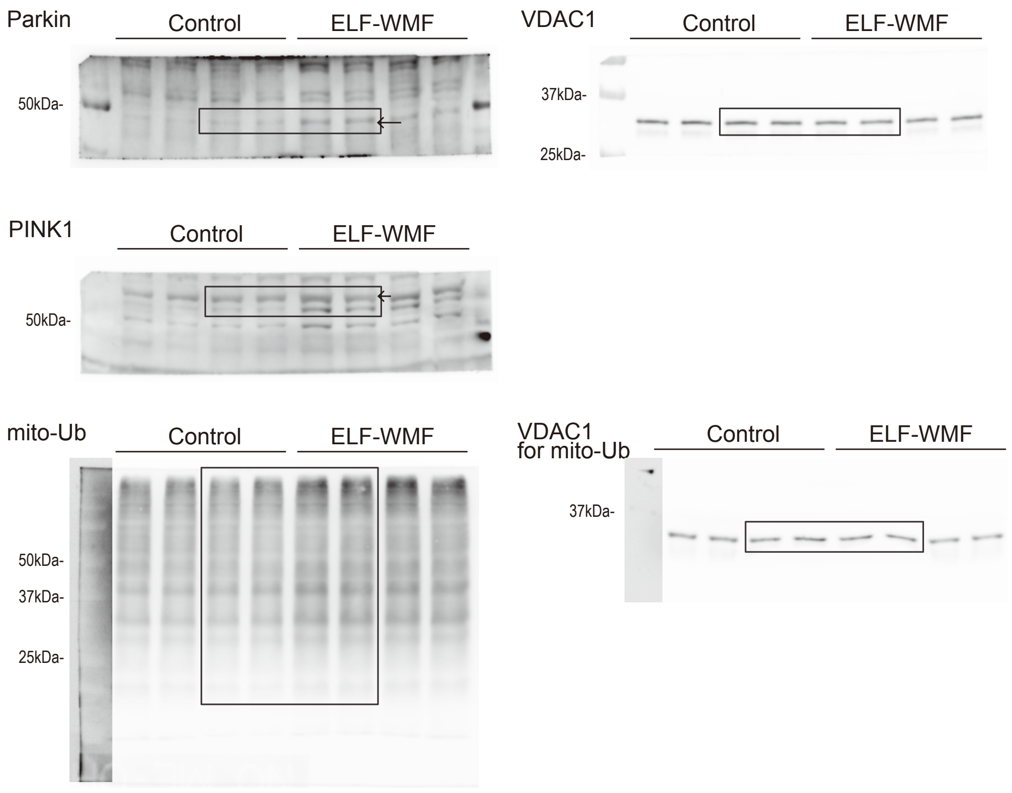

Fig.5

PGC-1a

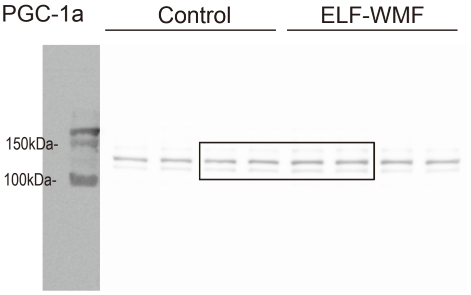

PPARa

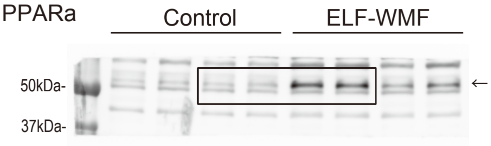

TFAM

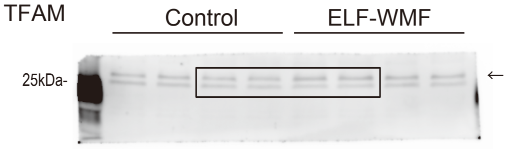

b-actin

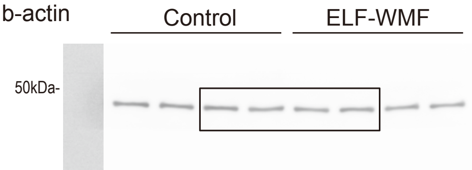

Fig.S3

OXPHOS

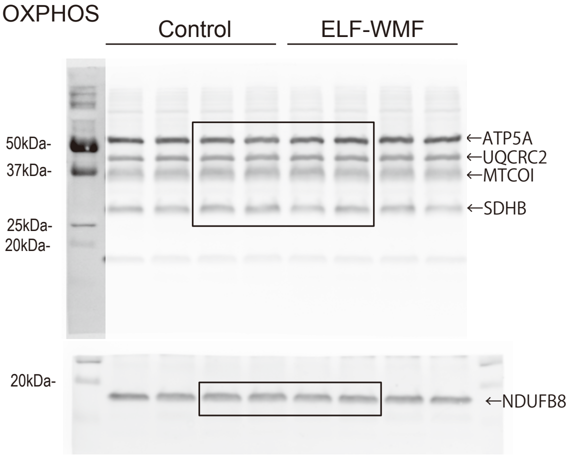

b-actin
for OXPHOS

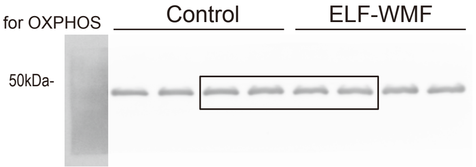

VDAC1

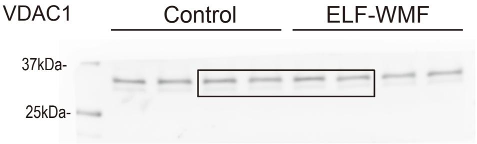

b-actin
for VDAC1

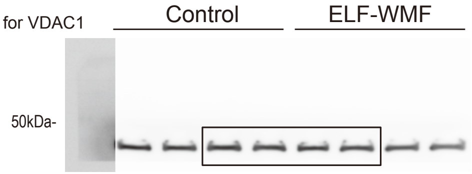

Fig.S4b

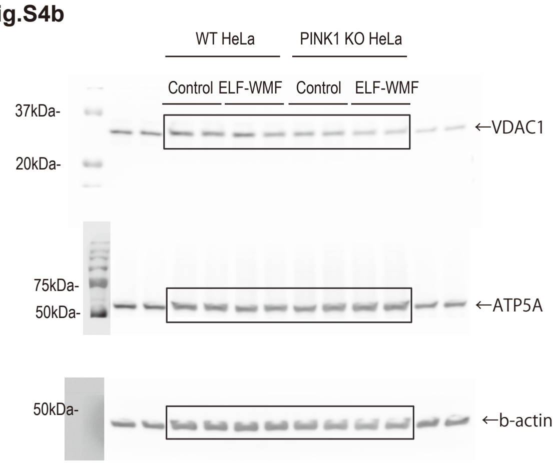

Fig.S5

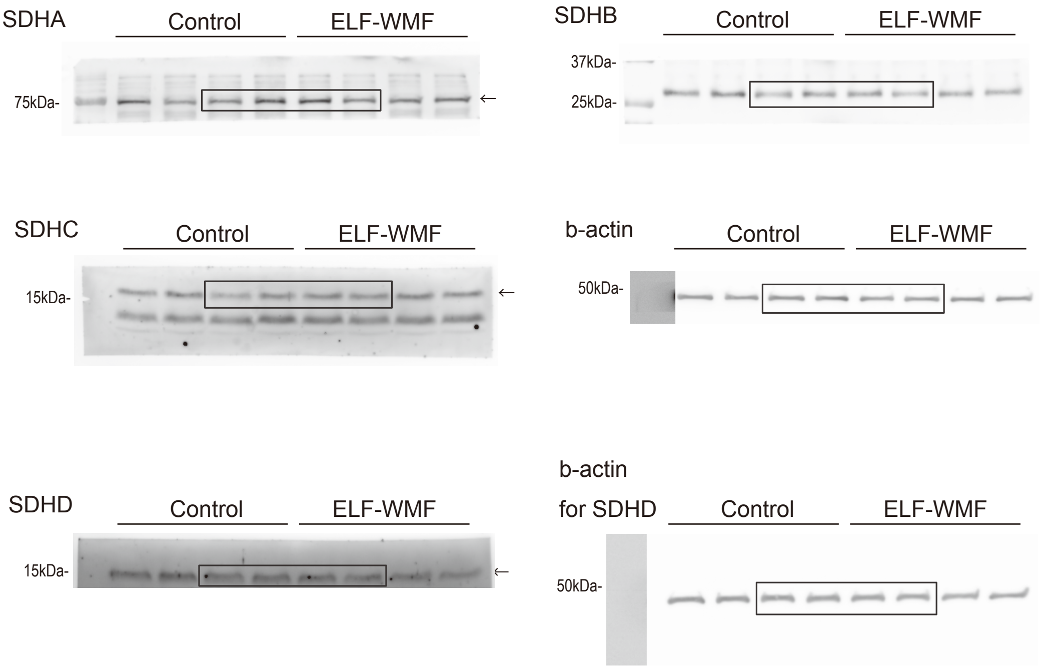

Supplement: Supplementary file 2 — Supplementary Information [file 42003_2022_3389_MOESM2_ESM.pdf]
