# Supplementary material for: Extremely low-frequency pulses of faint magnetic field induce mitophagy to rejuvenate mitochondria
Source: Commun Biol. 2022 May 12;5:453. doi: 10.1038/s42003-022-03389-7 (PMC9098439; doi:10.1038/s42003-022-03389-7)
Supplement: Supplementary file 5 — Reporting Summary [file 42003_2022_3389_MOESM5_ESM.pdf]

nature research

Corresponding author(s): Kinji Ohno

Last updated by author(s): Feb 13, 2022

Reporting Summary

Nature Research wishes to improve the reproducibility of the work that we publish. This form provides structure for consistency and transparency in reporting. For further information on Nature Research policies, see our Editorial Policies and the Editorial Policy Checklist.

Statistics

For all statistical analyses, confirm that the following items are present in the figure legend, table legend, main text, or Methods section.

n/a	Confirmed	
☐	☒	The exact sample size (n) for each experimental group/condition, given as a discrete number and unit of measurement
☐	☒	A statement on whether measurements were taken from distinct samples or whether the same sample was measured repeatedly
☐	☒	The statistical test(s) used AND whether they are one- or two-sided<br>*Only common tests should be described solely by name; describe more complex techniques in the Methods section.*
☐	☒	A description of all covariates tested
☐	☒	A description of any assumptions or corrections, such as tests of normality and adjustment for multiple comparisons
☐	☒	A full description of the statistical parameters including central tendency (e.g. means) or other basic estimates (e.g. regression coefficient) AND variation (e.g. standard deviation) or associated estimates of uncertainty (e.g. confidence intervals)
☐	☒	For null hypothesis testing, the test statistic (e.g. F, t, r) with confidence intervals, effect sizes, degrees of freedom and P value noted<br>*Give P values as exact values whenever suitable.*
☒	☐	For Bayesian analysis, information on the choice of priors and Markov chain Monte Carlo settings
☒	☐	For hierarchical and complex designs, identification of the appropriate level for tests and full reporting of outcomes
☒	☐	Estimates of effect sizes (e.g. Cohen's d, Pearson's r), indicating how they were calculated

Our web collection on statistics for biologists contains articles on many of the points above.

Software and code

Policy information about availability of computer code

Data collection	Not applicable.
Data analysis	Not applicable.

For manuscripts utilizing custom algorithms or software that are central to the research but not yet described in published literature, software must be made available to editors and reviewers. We strongly encourage code deposition in a community repository (e.g. GitHub). See the Nature Research guidelines for submitting code & software for further information.

Data

Policy information about availability of data

All manuscripts must include a data availability statement. This statement should provide the following information, where applicable:

- Accession codes, unique identifiers, or web links for publicly available datasets
- A list of figures that have associated raw data
- A description of any restrictions on data availability

We indicated in Materials and Methods that RNA-seq data were deposited in the Gene Expression Omnibus with an accession number GSE166811.

Field-specific reporting

Please select the one below that is the best fit for your research. If you are not sure, read the appropriate sections before making your selection.

☒ Life sciences ☐ Behavioural & social sciences ☐ Ecological, evolutionary & environmental sciences

For a reference copy of the document with all sections, see nature.com/documents/nr-reporting-summary-flat.pdf

Life sciences study design

All studies must disclose on these points even when the disclosure is negative.

Sample size	This report is an exploratory research with wild-type mice.
Data exclusions	No data were excluded from the analysis.
Replication	All the mouse studies were performed in 14 mice in each group. We indicated the number of mice for each figure legend.
Randomization	Mice were randomly divided into the treated and untreated groups.
Blinding	No subject measures were employed in the current studies, and experimental data were not blinded.

Reporting for specific materials, systems and methods

We require information from authors about some types of materials, experimental systems and methods used in many studies. Here, indicate whether each material, system or method listed is relevant to your study. If you are not sure if a list item applies to your research, read the appropriate section before selecting a response.

Materials & experimental systems

n/a	Involved in the study	
☐	☒	Antibodies
☐	☒	Eukaryotic cell lines
☒	☐	Palaeontology and archaeology
☐	☒	Animals and other organisms
☒	☐	Human research participants
☒	☐	Clinical data
☒	☐	Dual use research of concern

Methods

n/a	Involved in the study	
☒	☐	ChIP-seq
☐	☒	Flow cytometry
☒	☐	MRI-based neuroimaging

Antibodies

Antibodies used	We showed identities of all antibodies in Materials and Methods.
Validation	All antibodies were commercial products. Validation data were provided by the manufacturers.

Eukaryotic cell lines

Policy information about cell lines

Cell line source(s)	We used six mammalian cell lines, and their sources were indicated in Materials and Methods.
Authentication	We only used established cell lines.
Mycoplasma contamination	We routinely examine the contamination by mycoplasma, and obtain negative results.
Commonly misidentified lines (See ICLAC register)	We confirmed that we did not use misidentified cell lines.

Animals and other organisms

Policy information about studies involving animals; ARRIVE guidelines recommended for reporting animal research

Laboratory animals	We described mouse strain, sex and age in Materials and Methods.

Wild animals	Not applicable.
Field-collected samples	Not applicable.
Ethics oversight	All animal studies were performed after approval by the Animal Care and Use Committee of Nagoya University. We also followed relevant guidelines.

Note that full information on the approval of the study protocol must also be provided in the manuscript.

Flow Cytometry

Plots

Confirm that:

☐ The axis labels state the marker and fluorochrome used (e.g. CD4-FITC).

☐ The axis scales are clearly visible. Include numbers along axes only for bottom left plot of group (a 'group' is an analysis of identical markers).

☐ All plots are contour plots with outliers or pseudocolor plots.

☒ A numerical value for number of cells or percentage (with statistics) is provided.

Methodology

Sample preparation	We described how we prepared samples in Materials and Methods.
Instrument	We showed the model name of the Flow Cytometry in Materials and Methods.
Software	We described the analysis software for Flow Cytometry data in Materials and Methods.
Cell population abundance	We counted more than 10,000 cells for each Flow Cytometry analysis.
Gating strategy	We filtered out dead cells and cellular debris.

☐ Tick this box to confirm that a figure exemplifying the gating strategy is provided in the Supplementary Information.
